# Personalized neuromusculoskeletal modeling to improve treatment of mobility impairments: a perspective from European research sites

**DOI:** 10.1186/1743-0003-9-18

**Published:** 2012-03-30

**Authors:** Benjamin J Fregly, Michael L Boninger, David J Reinkensmeyer

**Affiliations:** 1 Departments of Mechanical & Aerospace Engineering, Biomedical Engineering, and Orthopaedics & Rehabilitation, University of Florida, 231 MAE-A Building, P.O. Box 116250, Gainesville, FL 32611-6250, USA; 2 Department of Physical Medicine & Rehabilitation, University of Pittsburgh, Pittsburgh, PA, USA; 3 Departments of Mechanical & Aerospace Engineering and Biomedical Engineering, University of California, Irvine, CA, USA

**Keywords:** Musculoskeletal model, Neural control model, Orthopedic surgery, Neurorehabilitation, Biomechanics

## Abstract

Mobility impairments due to injury or disease have a significant impact on quality of life. Consequently, development of effective treatments to restore or replace lost function is an important societal challenge. In current clinical practice, a treatment plan is often selected from a standard menu of options rather than customized to the unique characteristics of the patient. Furthermore, the treatment selection process is normally based on subjective clinical experience rather than objective prediction of post-treatment function. The net result is treatment methods that are less effective than desired at restoring lost function. This paper discusses the possible use of personalized neuromusculoskeletal computer models to improve customization, objectivity, and ultimately effectiveness of treatments for mobility impairments. The discussion is based on information gathered from academic and industrial research sites throughout Europe, and both clinical and technical aspects of personalized neuromusculoskeletal modeling are explored. On the clinical front, we discuss the purpose and process of personalized neuromusculoskeletal modeling, the application of personalized models to clinical problems, and gaps in clinical application. On the technical front, we discuss current capabilities of personalized neuromusculoskeletal models along with technical gaps that limit future clinical application. We conclude by summarizing recommendations for future research efforts that would allow personalized neuromusculoskeletal models to make the greatest impact possible on treatment design for mobility impairments.

## Introduction

Mobility involves walking, stair climbing, posture, balance, manipulation, transfers, and other locomotion tasks and is therefore central to qualify of life. When an individual incurs a mobility impairment, quality of life is diminished in proportion to the extent of the impairment. For example, mild knee osteoarthritis can limit participation in desired recreational or athletic activities without significantly affecting normal daily activities and productivity. In contrast, a stroke can make it nearly impossible to walk or manipulate objects, significantly diminishing an individual's ability to be self sufficient and function in society. Spinal cord injury can leave a person with normal upper extremity function but no remaining lower extremity function, significantly impacting only certain aspects of mobility.

Treatments for different mobility impairments are typically stereotypical, with a standard menu of treatment options existing for any particular mobility impairment. For example, severe medial compartment knee osteoarthritis may be treated surgically using high tibial osteotomy, unicondylar knee replacement, or total knee replacement. Once a patient seeks surgical treatment for debilitating pain and significant loss of function, the clinician must choose between these treatment options based on clinical assessment of the patient. Furthermore, the clinician must determine the optimal values of the parameters associated with the selected treatment (e.g., method, level, and amount of correction for tibial osteotomy, and implant type, size, and positioning for joint replacement). A similar situation exists for rehabilitation and surgical treatments of neurological disorders such as stroke, Parkinson's disease, and cerebral palsy. In clinical practice, the final treatment plan is usually selected based on subjective clinical experience rather than on objective prediction of post-treatment function developed from patient data.

Personalized computational models of the neuromusculoskeletal system could facilitate objective prediction of patient-specific functional outcome for different treatment designs being considered by the clinician. Depending on the intended clinical application, a personalized neuromusculoskeletal model could account for patient-specific anatomical (e.g., skeletal structure and muscle lines of action), physiological (e.g., muscle force-generating properties), and/or neurological (e.g., constraints on achievable muscle excitation patterns) characteristics, all within the context of a multibody dynamic model. Personalized models for treatment design are motivated by the fact that for many treatments, "one size fits none." Every patient is different and possesses unique anatomical, neurological, and functional characteristics that may significantly impact optimal treatment of the patient. Personalized models provide one possible avenue for increased objectivity in treatment planning, reducing the likelihood that different clinicians will plan different treatments given the same patient data. Ideally, virtual treatments performed on a patient's personalized model would allow objective and reliable prediction of post-treatment function and thus identification of an optimal treatment plan. Such predictions would identify not only the best type of treatment (including previously unknown treatments) but also treatment parameters to which functional outcome is highly sensitive (i.e., which treatment parameter values does the clinician need to "get right"?).

This paper explores how personalized neuromusculoskeletal models could be used to improve treatment design for mobility impairments. The exploration is based on a survey of personalized modeling research being performed in Europe and thus is limited in its scope. The survey was funded by the National Science Foundation (NSF) in the United States with the goal of synthesizing research recommendations and informing research funding in the area of technology to improve mobility. In October of 2010, two teams of four panelists recruited by NSF visited a number of academic and industrial sites throughout Europe over a one week time period. Since time and financial constraints limited the number of labs that could be visited, it was not possible to gather information from all labs in Europe performing valuable work in this area. Given that the goal of the tour was to survey the state-of-the-art in Europe, we also omit discussion of valuable work being performed by labs outside of Europe. The remainder of this paper summarizes the panel's findings related to the potential clinical use and benefit of personalized neuromusculoskeletal modeling.

### Clinical aspects of personalized modeling

In this section, we discuss current and future clinical uses of personalized neuromusculoskeletal models to design improved treatments for mobility impairments. To set the stage, we begin by discussing common reasons why human movement data are collected, followed by a proposal for a general process to follow when using personalized models in the treatment design process. We then discuss mobility-related clinical problems currently being addressed with personalized neuromusculoskeletal models, and we conclude this section by highlighting gaps in clinical application where personalized models could add significant value.

#### Clinical purpose of personalized modeling

Pre-treatment human movement (e.g., motion capture, ground reaction, muscle electromyographic, energy consumption), strength (e.g., isometric and isokinetic dynamometer), and imaging (e.g., magnetic resonance (MR), computed tomography (CT), x-ray, fluoroscopic) data provide the experimental measurements necessary to develop objective model-based predictions of post-treatment function. As described by Dr. Maria Grazia Benedetti at the Rizzoli Orthopedic Institute in Bologna, Italy, there are three primary reasons for collecting human movement data in a clinical setting:

1) *Assessment *- Assess after treatment how the treatment worked for an individual patient or a group of patients. An example would be using gait data to assess changes in walking speed and knee flexion angle following tendon transfer or lengthening surgery in a specific child or group of children with cerebral palsy. This use of human movement data is relatively common.

2) *Identification *- Identify on an individual patient basis which patients should be treated (but not how they should be treated). An example would be using gait data to determine whether tendon transfer or tendon lengthening surgery should be performed for a specific child with cerebral palsy. This use of human movement data remains uncommon but is becoming more common.

3) *Prediction *- Predict on an individual patient basis which treatment should be performed and how it should be performed. An example would be using gait data to determine whether tendon transfer or tendon lengthening surgery should be performed, which tendon to transfer or lengthen, and where to transfer it or how much to lengthen it, to improve walking ability for a specific child with cerebral palsy. This use of human movement data does not yet happen in clinical practice.

The focus of this paper is on how personalized neuromusculoskeletal models could be used for *prediction *rather than *assessment *or *identification*, though *identification *has significant clinical value as well. While *prediction *is the most challenging use, it is also the use with the greatest potential to improve functional outcome on an individual patient basis.

#### Clinical process of personalized modeling

How should personalized neuromusculoskeletal models be used to predict functional outcome for various treatment plans under consideration? Expanded from ideas presented by researchers at the Rizzoli Orthopedic Institute in Bologna, Italy, and Dr. Bart Koopman at the University of Twente in Enschede, the Netherlands, we propose a three-step process for treatment design using personalized models:

1) *Model preparation steps:*

• **Identify **model outputs to be used as indicators of clinical/functional outcome.

• **Define **model complexity required to predict these outputs with sufficient accuracy for the intended clinical application.

• **Collect **pre-treatment movement, strength, and imaging data (as required) to construct the personalized model and predict the outputs of interest.

2) *Model construction steps:*

• **Calibrate **model geometry and parameter values to which the outputs of interest are sensitive using pre-treatment movement, strength, and/or imaging data.

• **Estimate **model parameter values to which the outputs of interest are insensitive using data reported in the literature.

• **Incorporate **surgical or rehabilitation treatment plans under consideration into the personalized model.

3) *Model utilization steps:*

• **Predict **post-treatment patient function for each proposed treatment plan.

• **Select **treatment plan and associated parameter values that maximize functional outcome, possibly using numerical optimization methods.

• **Validate **personalized model predictions using post-treatment function measured from patients whose treatment was not planned with a model.

• **Implement **optimal surgical or rehabilitation treatment plan designed with the personalized model.

• **Collect **post-treatment movement, strength, and/or imaging data from the patient to assess clinical/functional outcome.

In this process, only the steps relevant to the mobility-related clinical application at hand need be performed. For example, clinical applications that do not require modeling of individual muscle forces may not require any imaging and strength data from the patient, and thus steps related to calibration of patient-specific muscle and bone geometry can be omitted. Model parameter values that require calibration to patient data may necessitate collection of additional experimental data solely for calibration purposes [1].

Two critical tasks to highlight in this process are **Calibrate **and **Validate**. Unless the model is calibrated to relevant data collected from the patient prior to treatment, the model will not be sufficiently personalized to predict the patient's post-treatment function. Similarly, unless calibrated model predictions are validated using post-treatment data collected from patients whose treatments were not planned with the model, clinicians will not have confidence in the model predictions, and personalized models will never advance toward widespread clinical utility. Validation of treatment planning using personalized models will ultimately require randomized controlled trials, where outcomes are compared between patients whose treatments were planned with a personalized model and those whose treatments were not.

#### Clinical applications of personalized modeling

During our tour of European research labs, we sought to identify clinical applications where a personalized modeling process similar to the one outlined above was already being followed. By the end of the tour, we made three valuable observations related to clinical application of personalized neuromusculoskeletal models. First, few labs have reached the point of being able to apply this process to specific clinical problems. Second, some of the best existing clinical applications involved generic rather than personalized models. Third, most clinical applications we observed involved orthopedic surgery, with few applications involving neurorehabilitation. Below we comment further on these observations.

Three large projects funded by the European Commission (EC) are making significant strides in developing and applying personalized neuromusculoskeletal models to orthopedic clinical problems. The first is the "Osteoporotic Virtual Physiological Human" (VPHOP) project [2], which involves a large consortium of academic and industrial partners throughout Europe and is coordinated by Dr. Marco Viceconti at the Rizzoli Orthopedic Institute in Bologna, Italy [3-5]. As stated on the project website, the goal is to "develop, validate and deploy the next generation of technology to predict the absolute risk of fracture in patients with low bone mass, thereby enabling clinicians to provide better prognoses and implement more effective treatment strategies." One of the unique emphases of the project is on multi-scale modeling, with bone being modeled simultaneously on the cell, tissue, organ, and body levels to permit clinically useful predictions of the risk of bone fracture in different patient populations.

**Figure 1:**
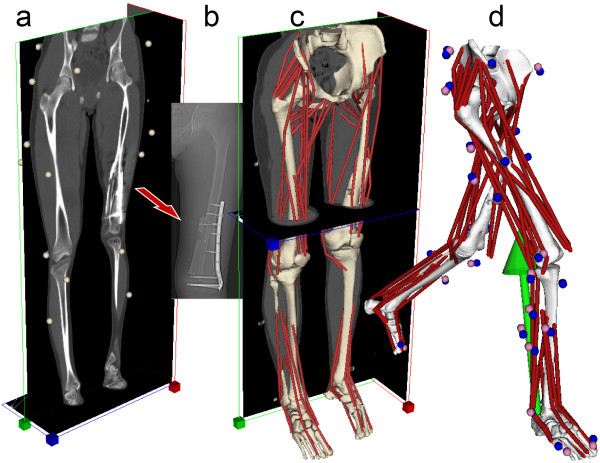
**Personalized modeling workflow for massive skeletal reconstruction, as developed by the Medical Technology Laboratory at the Rizzoli Orthopedic Institute in Bologna, Italy**. a) CT scan of the lower limbs performed at follow-up, with motion capture markers visible as well. b) Focused view of reconstructed femur immediately after surgery. c) Patient-specific musculoskeletal model superimposed on CT images (LHPBuilder, B3C, Italy). d) One frame of dynamic walking simulation performed with the patient-specific model (OpenSim). Image courtesy of Dr. Giordano Valente, Rizzoli Orthopedic Institute, Bologna, Italy.

In a related project, Viceconti's team at the Rizzoli Orthopedic Institute has developed personalized neuromusculoskeleal models of pediatric patients who received a surgical limb salvage procedure for bone cancer [4,6]. For this clinical problem, the challenge is to determine how the patient should load the bone allograft during the rehabilitation process such that bone loads are high enough to stimulate repair but low enough to avoid fracture. Since each clinical case is unique, surgical and rehabilitation treatment design cannot be standardized. Dr. Viceconti and his research team are using gait and imaging data to create personalized neuromusculoskeletal models that estimate muscle and bone loads in the patient's femur during walking (Figure 1). These estimates inform the rehabilitation process and when the patient should be cleared for full functional loading with no restrictions. The primary challenges faced by this personalized modeling process are whether scaling of muscle and bone geometry from a generic model is sufficiently accurate for this pediatric application, and also whether the estimated muscle and bone loads (which currently cannot be validated experimentally) are sufficiently reliable.

The second large EC-funded project is called "NMS Physiome" [7], which is also coordinated by researchers at the Rizzoli Orthopedic Institute. This project seeks to "promote a more organic cooperation in the development of Predictive, Personalised and Integrative musculoskeletal medicine" by integrating research efforts between the VPHOP project and the Center for Physics-based Simulation of Biological Structures (Simbios) at Stanford University in the United States. Integration is focused on neuromusculoskeletal software tools (MAF, OpenSim, and FEBio) and research community websites (BiomedTown and Simtk) developed by the two consortia. The goal of integration is to address the challenges posed by personalized neuromusculoskeletal modeling more effectively and efficiently.

**Figure 2:**
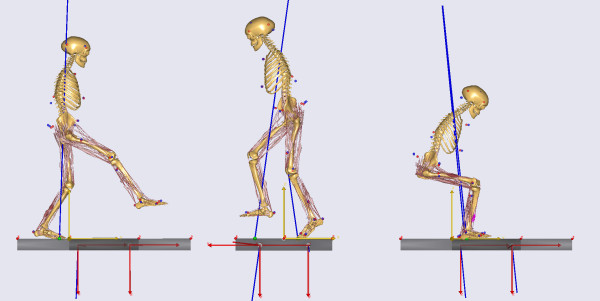
**Examples of musculoskeletal models developed for the TLEMsafe project**. Image courtesy of Prof. Dr. Ir. Bart Koopman, University of Twente, Enschede, the Netherlands.

The third large EC-funded project is entitled "Improving Safety and Predictability of Complex Musculo-skeletal Surgery using a Patient-Specific Navigation System" (TLEMsafe) [8], which involves a consortium of academic and industrial partners headed by the University of Twente in Enschede, the Netherlands. The stated goal of the project is to "create an ICT-based patient-specific surgical navigation system that helps the surgeon safely reach the optimal functional result for the patient and is a user friendly training facility for the surgeons." In this project, the researchers proposed to use personalized neuromusculoskeletal models as part of a three-step treatment design process. The first step is creation of the personalized model from the patient's movement and imaging data. The model is created within the framework of the AnyBody musculoskeletal modeling software developed by researchers at the University of Aalborg in Denmark [9] (Figure 2). The second step is for the surgeon to perform virtual surgical treatments on the personalized model and to identify the optimal surgical plan for the patient. The final step is to transfer the optimized treatment plan into a surgical navigation system to be used during actual surgery. As part of this project, Dr. Bart Koopman of the University of Twente is currently investigating the use of personalized neuromusculoskeletal models to identify optimal patient-specific tendon transfer procedures to restore hip adductor strength in patients who walk with a "drooping" swing leg hip (i.e., Trendelenburg gait due to Poliomyelitis or total hip arthroplasty).

Other research we observed involved the use of generic rather than personalized neuromusculoskeletal models. While personalized models have the greatest potential to impact clinic practice, generic models can still provide significant clinical benefits. Two clinical applications of generic models were particularly well developed. The first was the design of a total ankle replacement by Dr. Alberto Leardini and colleagues at the Rizzoli Orthopedic Institute in Italy [10,11]. The design was developed using a sagittal plane musculoskeletal model of the ankle that incorporated the ligaments and articular surfaces. The design philosophy was to maintain medial and lateral ankle ligaments in an isometric state during passive ankle motion. The team identified a novel geometric design to achieve this goal, using non-anatomically shaped tibial and talar components with a meniscal bearing interposed between them. The design has been licensed by an orthopedic implant company, and early clinical assessment is demonstrating good restoration of ankle mobility with low complication and revision rates [12,13].

The other example was evaluation of tendon transfer surgery for massive rotator cuff tears by Dr. Frans van der Helm and colleagues at Delft University of Technology in the Netherlands [14,15]. The evaluation was performed using a high fidelity musculoskeletal shoulder and elbow model constructed from extensive measurements performed on a single cadaver specimen [16]. The model accounts for more parameters (including muscle sarcomere length measured by laser diffraction) than any other upper extremity model. Simulations performed with the generic model have provided specific recommendations for which tendons to transfer, and where to transfer them, to replace the function of a torn rotator cuff without sacrificing shoulder strength for functional tasks. Similar to the pediatric oncology application, the reliability of the model's predicted clinical outcome is only as good as the reliability of the model's predicted muscle forces. Dr. van der Helm and colleagues have attempted to validate the model's prediction of shoulder muscle and contact forces using contact forces measured by an instrumented shoulder prosthesis [17]. The authors concluded that, "Although results indicated a reasonable compatibility between model and measured data, adjustments will be necessary to individualize the generic model with the patient-specific characteristics."

The primary rehabilitation applications we observed utilized a personalized modeling method called Complementary Limb Motion Estimation (CLME) [18,19]. The method uses motion measurements made on a patient's healthy leg to predict how the patient's impaired leg should move. The predictions are made in real time by exploiting the strong coupling that exists between skeletal degrees of freedom during locomotion. These couplings (or synergies) are identified in healthy subjects using statistical dimensionality reduction methods (e.g., principal component analysis) and then applied to the healthy limb of a patient to predict the desired motion of the patient's impaired limb. CLME was first proposed to generate personalized motion trajectories for the paretic limb of patients undergoing robotic gait training following stroke [19]. The goal was to maintain patient stability while minimizing unwanted interaction forces between patient and robot. More recently the same method has been proposed to personalize the control of an active knee exoprosthesis to the gait patterns of patients who have undergone above-knee amputation [18]. For both applications, the predicted motion of the impaired or prosthetic limb is used as a personalized reference to be tracked by the robot or prosthesis control system. While we observed other personalized rehabilitation applications, they did not have a strong neuromusculoskeletal modeling component to them.

#### Clinical gaps in personalized modeling

Mobility-related clinical problems are typically treated by either surgery or rehabilitation and either do or do not possess a significant neurological component. Thus, use of personalized neuromuscular models to improve treatment of mobility impairments can be grouped into four categories: 1) surgical treatment without a significant neurological component, 2) surgical treatment with a significant neurological component, 3) rehabilitation treatment without a significant neurological component, and 4) rehabilitation treatment with a significant neurological component. Of these four categories, the first is the most developed and the fourth the least developed in terms of personalized neuromusculoskeletal modeling. This situation is not surprising given that technology is a more recent addition to rehabilitation treatments (e.g., rehabilitation robotics) than to surgical treatments and that neurological factors are more difficult to model than are mechanical factors. Below we present clinical gaps in personalized modeling for each of these four categories.

Osteoarthritis is a prevalent disabling disease that is commonly treated by surgical intervention. Though it clearly possesses a neurological component [20], that component is secondary to mechanical factors as far as surgical treatment design is concerned. Use of personalized neuromusculoskeletal models has been proposed by European labs for pre-operative planning of high tibial osteomities and total joint replacements [21,22]. While joint replacement surgery is generally reliable, individual cases can pose special challenges, especially those involving revision surgery. In contrast, high tibial osteotomy (HTO) is a challenging surgical procedure with highly variable outcomes but also high potential benefits, making it an excellent target for personalized models. Use of personalized models to design customized gait modifications following HTO surgery could also be valuable for avoiding the loss of boney correction that often occurs over time. Anterior cruciate ligament replacement to avoid knee osteoarthritis is a related surgical application where personalized models could be of value [23].

In contrast, cerebral palsy and Charcot-Marie-Tooth disease are neurological disorders that are commonly treated by surgery, since no treatment exists for the underlying neurological problem. Though Charcot-Marie-Tooth disease is not well known, it is the most commonly inherited neurological disorder and limits mobility in approximately 1 in 2,500 individuals [24]. Surgical treatments for both disorders typically involve muscle lengthening, tendon transfer, and/or osteotomy to improve joint range of motion, foot-ground contact pattern, gait speed, and gait symmetry. For both disorders, patients have varied and unique clinical presentations, making stereotypical treatment planning ineffective. For this reason, personalized neuromusculoskeletal models, especially those that are able to model the neurological limitations (e.g., muscle spasticity) of the patient, could play a valuable role in predicting the outcome of complex multi-level surgeries that are performed on these patients [25-27].

Stroke, spinal cord injury, and traumatic brain injury significantly affect mobility, possess a major neurological component, and are often treated by rehabilitation methods. Personalized neuromusculoskeletal models have yet to be applied to traditional or robot-assisted rehabilitation treatments for these disorders. Given this large gap, even personalized models that omit neural control models have the potential to make a significant clinical impact. For example, a number of clinical and research labs in Europe are utilizing robot-assisted therapy for neurorehabilitation [28,29]. Many of these labs are using the Lokomat gait trainer (Hocoma AG, Volketswil, Switzerland), whose programmed walking pattern is that of one of the designers. Personalized musculoskeletal models that can predict patient-specific improvements in gait pattern could be used to customize robot-prescribed gait motions. For individuals who have had a stroke, similar models could be used to predict a patient-specific sequence of gradual gait alterations leading to normal function, with the model indentifying where to focus rehabilitation efforts to maximize functional outcome.

Personalized musculoskeletal models that include personalized neural control models would be even more beneficial for improving rehabilitation of neurological disorders. Models that account for patient-specific neural control limitations and neuroplasticity could be used to identify the maximum expected improvement and how best to get there. As suggested by Dr. Herman van der Kooij of the University of Twente in the Netherlands, such models could be useful for predicting how people interact with and adapt to their environments, which could improve the effectiveness of robotic therapy systems. Furthermore, such models could be valuable for the design of neuroprostheses that use functional electrical stimulation to restore lost function [30,31]. For example, if a personalized neuromusculoskeletal model could predict a minimum set of muscles to stimulate, and how and when to stimulate them, to restore a normal gait pattern, then the personalized prescription could be investigated in a clinical environment. As stated by one of the researchers on our panel, "There is a need for. . . improved models of human motor recovery to provide a more rational framework for designing robotic therapy control strategies." [32].

One of the primary reasons for these clinical gaps is lack of effective collaboration between clinical researchers and personalized modeling researchers. An excellent counterexample is the Rizzoli Orthopedic Institute in Italy, where clinicians and engineers share the same office space and interact during clinical decision making. These interactions create an atmosphere where clinicians routinely enter into the technical world and engineers routinely enter into the clinical world. Such an environment of shared intellectual investment in solving clinical problems is critical if personalized neuromusculoskeletal modeling is to make a broad impact in the clinic.

### Technical aspects of personalized modeling

Significant research efforts are currently underway in labs throughout Europe to develop personalized neuromusculoskeletal modeling tools and methods. Recalling the section above on the *Clinical Process of Personalized Modeling*, the primary challenges faced by these efforts are the **Calibrate **step within *Model construction *and the **Validate **step within *Model utilization*. In this section, we discuss current technical capabilities of personalized modeling related to model calibration and validation, followed by a discussion of technical gaps that need to be filled if personalized neuromusculoskeletal models are to become clinically useful.

#### Technical capabilities of personalized modeling

Despite significant computational advances over the past ten years, model personalization remains a major challenge, as does the ability to use a personalized model to predict the outcome of a clinical intervention. Personalized neuromusculoskeletal models can be applied to mobility-related clinical problems only to the extent to which key model features can be calibrated to data collected from a patient. Thus, the ability to calibrate models to patient data is a prerequisite to clinical use of personalized models, with the proposed clinical application determining the extent of model personalization required.

Since most neuromusculoskeletal models are generic, being constructed from detailed anatomic measurements performed on cadaver specimens [16,33], a model personalization (or calibration) process is needed. Expanding on information provided by Dr. Bart Koopman of the University of Twente in the Netherlands, we propose four model calibration steps that should be performed in whole or in part to transform a generic model into a personalized model:

1) *Geometric calibration *- Use of imaging data (e.g., MR, CT, x-ray) to calibrate bone geometry, muscle lines of action, and muscle moment arms in a musculoskeletal model.

2) *Kinematic calibration *- Use of motion data (e.g., marker-based, inertial sensors, fluoroscopy) to calibrate constraint-based joint positions and orientations in the body segments of a skeletal model.

3) *Kinetic calibration *- Use of load data (e.g., ground reaction force and moment, foot contact pressure, dynamometer) to calibrate body segment mass and inertia, foot stiffness, muscle strength, and other muscle-tendon properties in a neuromusculoskeletal model.

4) *Neurologic calibration *- Use of motion, load, and muscle activity data (i.e., muscle EMG) to calibrate feedforward, intrinsic feedback, reflexive feedback, and/or synergy properties of the neural control system in a neuromusculoskeletal model.

The challenge is how to construct a personalized model that is consistent with all available data from these different modalities [34].

Current methods for geometric calibration involve uniform scaling, non-uniform scaling, deformation, or direct creation of bone models and muscle lines of action from patient MR or CT data. Uniform scaling based on external measurements is inaccurate when calculating muscle moment arms, muscle-tendon lengths, muscle forces, and joint contact forces [35], especially when scaling a generic model of an adult to a pediatric patient [36]. Non-uniform scaling is only slightly better at producing accurate muscle moment arms and muscle-tendon lengths [27]. Creation of patient-specific geometry directly from the patient's imaging data remains the gold standard [26], but the process is highly time consuming and somewhat subjective, depending on the imaging modality and the anatomic structures being modeled (e.g., bone edges are often poorly defined in MR data).

Kinematic calibration involves determining fixed joint positions and orientations in the body segments of a skeletal model with a pre-defined kinematic structure. The calibration process is usually performed using surface marker data, with joint angles in the model being calculated as a byproduct. European labs have used optimization methods [1] and extended Kalman filter methods [37-39] to perform kinematic calibration. Filter-based methods have the advantage of being computationally faster and less complex than most optimization methods, but they require a greater amount of algorithm tuning to achieve satisfactory performance. Despite these advances, neither approach has yet to be generalized and incorporated into commercial-grade musculoskeletal modeling software such as the AnyBody program.

Kinetic calibration typically involves calibration of segment mass properties or muscle force-generating properties. Though segment mass properties can be calibrated to force plate and motion data [40], they are often taken from regression equations developed from measurements performed on cadavers [41]. Similarly, though muscle model parameter values (e.g., muscle strength) can be calibrated to isometric and/or isokinetic dynamometer data [42], this calibration step is usually omitted due to the extra effort it requires. A recent European study indicated that subject-specific muscle-tendon parameter values calibrated to dynamometer data are appropriate for use in musculoskeletal models used to analyze gait [43]. Since many movement impairments involve undesirable foot-ground contact patterns, kinetic calibration of patient-specific foot-ground contact models will be essential in the future for predicting changes in gait function due to various proposed treatments, yet kinetic calibration methods for such models do not yet exist.

A promising development to address geometric, kinematic, and kinetic calibration simultaneously is research being performed by Dr. Wafi Skalli at Arts et Métiers ParisTech in Paris, France using the EOS bi-plane x-ray system (EOS Imaging SA, Paris, France). With the EOS system, a subject stands upright while a low-dose x-ray system scans the entire body from head to toe collecting one continuous distortion-free image in each of two orthogonal planes. The resulting bi-plane images are then processed and morphed using template anatomy for personalization and visualization. Geometric calibration of bone and muscle geometry via deformation of template bone and muscle models can be performed rapidly and accurately relative to geometry constructed directly from CT data [44-47]. Kinematic calibration could theoretically be aided by performing scans of the relevant portion of the body in two or more poses (e.g., the lower extremities in different squatting positions) [48], especially if surface markers to be used in additional movement experiments are also visible. Kinetic calibration of segment mass properties and muscle strength parameters (based on muscle cross sectional areas) can also be performed from the images [49,50]. Thus, with improvements in automation and refinement of existing algorithms, the EOS system has the potential to improve musculoskeletal model personalization significantly.

The remaining area, neurologic calibration, has seen the least progress due to the significant challenges involved in understanding how the human neural control system functions. This calibration step can be pursued using a range of approaches, from a physiological approach that seeks to model the detailed anatomy and physiology involved in neural control, to an emergent approach that seeks to model the neural control computations implemented by the anatomy but without modeling anatomic detail. An example of a physiological approach is detailed modeling of feedforward, intrinsic (i.e., muscle) feedback, and reflexive (i.e., visual, proprioceptive, and vesitibular) feedback mechanisms utilized by the neural control system [51-54]. To date, such high fidelity neural control models have been applied to postural control rather than movement tasks, and methods for personalizing the parameter values in these models are not yet well developed. At the other extreme, an example of an emergent approach is muscle synergy analysis, where EMG signals from a large number of muscles (e.g., 16) are decomposed into a smaller number of basis activation signals (e.g., 5) for all muscles plus a unique set of weights (often termed "modules") for each muscle that scale the activation signals [55,56]. Synergy analysis is used for dimensionality reduction (e.g., 5 basis signals are used to reconstruct 16 EMG signals) and can identify neural control limitations in patients following stroke [57]. Incorporation of these limitations into personalized neuromusculoskeletal models could facilitate prediction of best possible functional outcome. Between these two extremes is a physiological approach that has successfully explained motor learning using simplified feedforward and feedback models [58]. The approach uses a v-shaped learning function to model the change in muscle feedforward commands generated in response to kinematic errors experienced during the previous movement trial. Computer simulation of a sequence of arm movement trials performed in different force fields revealed that the method can successfully reproduce experimentally observed trial-to-trial changes in muscle activations (to control force) and co-contraction (to control impedence).

Validation of clinical predictions is the other major challenge faced by personalized models. This challenge can be surmounted when clinical outcome variables are external quantities that can be easily measured (e.g., gait speed, gait symmetry). Frequently, however, established clinical databases use coarse ordinal scales to rank movement ability, and mapping these scores to neuromechanical models is difficult. In addition, significant challenges remain when the outcome variables are either internal to the body (e.g., muscle forces, joint contact forces, bone strains) or dependent on quantities that are internal to the body. Since such quantities cannot be measured directly by non-invasive means, alternate methods are needed for personalized model validation. For example, predicted muscle forces have been evaluated indirectly using in vivo joint contact force measurements [17] or novel measurements (e.g., near infrared spectroscopy) that are likely to be highly correlated with in vivo muscle force [59].

#### Technical gaps in personalized modeling

As suggested by this review of current technical capabilities in Europe, at least four critical technical gaps currently exist that limit the potential clinical applicability of personalized neuromusculoskeletal models:

1) *How can we make the personalized model calibration and prediction process fast and easy?*

Though several excellent musculoskeletal modeling programs exist, none of them contain functionality that automates the model calibration process and simplifies the model prediction process. Personalized model calibration and prediction currently require significant expertise and programming ability possessed by only a small number of researchers in a limited number of research labs. Making these capabilities available to the larger neuromusculoskeletal modeling community via fast, automated algorithms will be essential for the growth of personalized modeling efforts. Ultimately, personalized modeling will be adopted for routine clinical use only when it is extremely easy to use.

2) *How can we calibrate "unobservable" parameters to which model predictions are sensitive?*

For some clinical problems, personalized model predictions of functional outcome will be sensitive to model parameter values that cannot be calibrated to available data. The first step in addressing this problem is identifying when it occurs, which requires performing sensitivity analyses that in some cases will be limited by existing computational capabilities. The next step is development of new experimental methods or hardware that provide sufficiently rich information to calibrate the parameter values needed to develop the predictions.

3) *How can we create personalized neural control models?*

Few neuromusculoskeletal models published to date account for any level of personalized neural control modeling. Such modeling would ideally account for limitations in a patient's neural control capabilities as well as the extent of possible plasticity. Emergent approaches for modeling neural control could be incorporated into personalized musculoskeletal models as a starting point, while physiological models could be refined to the point where essential model parameters become well defined and methods for calibrating them are developed. The ability to incorporate complex personalized neural control models into personalized musculoskeletal models would greatly expand model applicability to clinical situations, especially those involving neurorehabilitation.

4) *How can we validate model-based predictions, especially for internal quantities such as muscle, joint, and bone loads?*

Validation of internal quantities that influence treatment design remains a major challenge. While researchers continue to refine optimization and EMG-driven methods for predicting muscle forces and related joint and bone loads, the ability to validate these predictions has lagged behind. Direct measurement of internal quantities under special conditions (e.g., instrumented implants), and the opportunity to test model-based predictions against these internal measurements, provides a valuable avenue for model validation efforts [60]. Identification of novel approaches that utilize only existing data collection capabilities, as well as development of new experimental techniques, will be essential if clinicians are to gain confidence in treatment plans designed with personalized neuromusculoskeletal models.

## Conclusions

Neuromusculoskeletal modeling has yet to make a significant difference in routine clinical practice. For this situation to change, the key gaps identified above need to be addressed by modeling researchers in close collaboration with clinical investigators. While the biggest clinical gap for personalized neuromusculoskeletal modeling is in neurorehabilitation, the gap for other mobility-related clinical problems is almost as large. The biggest technical gap is in personalized neural control and recovery models, though issues like automation of the model personalization process and development of personalized foot-ground contact models are critical as well for advancement. For clinical problems that involve highly unique patient characteristics, stereotypical treatment design is likely to yield variable functional outcomes. These types of clinical problems are where personalized neuromusculoskeletal models have the greatest potential to create a positive paradigm shift in the treatment design process.

## Competing interests

The authors declare that they have no competing interests.

## Authors' contributions

BF organized and drafted the manuscript based on information gathered during the European tour. ML provided clinical perspective on information gathered. DR organized the tour and the selection of European sites. All authors made significant contributions to gathering, critically evaluating, organizing, and revising the content of the manuscript. All authors read and approved the final manuscript.
